# Practical Notes and Formulæ

**Published:** 1878-01-20

**Authors:** 


### *PRACTICAL NOTES AND FORMULÆ.*

NOTE FROM DR. O. E. NEWTON ON HOW TO GIVE PODOPHYLLIN.

*Editor Southern Medical Record:*

I see on page 308 of your November issue, of last year, a suggestion how to give podophyllin, claiming it as a dangerous drug. There are but few physicians who know how to properly prepare podophyllin for use. I order the podophyllin as bought and sold to be put into a wedgewood mortar and most thoroughly triturated with equal parts of white sugar until the compound becomes impalpable, as the podophyllin, if given as bought and sold in its crude state, is almost sure to nauseate and gripe in full doses. I think if the auther of that article will have prepared podophyllin in the way I have suggested, he will find it to be generally free from the nauseating and griping tendency of which he complains.

As a compound podophyllin pill I use—

| | | |
|---|---|---|
| R.— | Triturated podophyllin and sugar | dr. i. |
| | Leptandria | dr. i. |
| | Rhei pulvis | dr. iss |
| | Capsici pulvis | grs. xv. |
| M. | Ext. zinziberis q. s. ut fiat pillulaes | 90 |

Dose, two to three pills after supper; if they do not move the bowels satisfactorily in twelve to fourteen hours, two more are to be given.

I have used millions of these pills with satisfactory results.

Podophyllin, as bought and sold from the manufacturers, is not fit to be used at any time, or for any purpose, in my opinion, though I frequently use it every day as above directed. A remedy that is so universally useful, and recognized as being useful wherever medicine is used throughout civilization, should be known by all how to use it.

CINCINNATI, January, 1878.

PERMANENT CURE FOR COSTIVENESS.

| | | |
|---|---|---|
| R— | Sodæ Sulphatis | grs. xx |
| | Ac. Nitro-muriat | gtt. v |

Take one hour before breakfast in half glass of water.—*Ib.*

NOTE FROM DR. O. E. NEWTON ON REMOVAL OF PLACENTA.

*Editor Southern Medical Record:*

SIR—I copy from page 288 of your November number, of last year, the following:

"With regard to the delivery of the placenta, the sooner you can make the patient comfortable the better. Therefore, as soon as you have cut the cord, which you may do as soon as respiration is well established, make traction upon the cord with one hand, and with the other grasp the uterus through the abdominal walls, and, as it were, squeeze out the placenta."

The plan I have adopted and continued to practice for the last twenty-five years is, as soon as I am ready to have the placenta pass, to take the cord in my left hand, trace it with my right fore-finger, passing on until I reach the external wall of the placenta, and with the corner of my right finger-nail puncture the membrane, when at once the placenta is relieved of its size by discharging the engorgement or accumulated venous blood, and will at once collapse. Then, by slight traction with the left hand, holding the cord, and the point of the right finger holding on to the placenta, it is easily drawn away. I never fail to remove the after-birth at once in this way, without there be present morbid adhesion of the afterbirth to the wall of the womb.

CINCINNATI, January, 1878.

PARALYSIS OF DIPHTHERIA.

Prof. Metcalfe, of New York, recommends the following prescription for the partially paralyzed, prostrated condition which sometimes succeeds an attack of diphtheria:

| | | |
|---|---|---|
| R— | Strychnine | gr. j. |
| | Dilute nitric acid | dr. j. |
| | Water | dr. vij. |

M. From three to five drops in a teaspoonful of water three times per day.

### PSEUDO HYPERTROPHIC PARALYSIS.

This affection is thus described in *Tanner's Index :*

"A disease of early childhood, mostly affecting males. The child weak on its legs, constantly falling, and getting up with difficulty; walk slow, clumsey and waddling. Great aching of loins. The calves of legs and buttocks become enlarged. No treatment effectual. Death usually occurs before the age of eighteen, from pulmonary consumption."

A condition resembling this not unfrequently occurs from the reflex irritation of worms in alimentary canal, which is promptly relieved by vermifuges. The following is excellent:

R—Fluid Ext. pink root
Syrup of Rhubarb..............aa—oz, j

Dose, one teaspoonful morning, noon and night. Into the last dose, at bedtime, add—

R—Calomel......................grs. v.

Santonine......................grs. j.

followed by dose of castor oil before breakfast next morning.

The worms being removed, use as a tonic for a few days—

R—Dialysed iron.................. dr. ii.

Tinc. nux vomica.............. dr. i.—M.

Dose five drops, to be given three times a day, for a child three to five years old.

### NIGHT CRAMP.

Cramps in the legs at night usually result from indigestion, attended with an excess of acid in the stomach and bowels. Persons subject to cramps should take light suppers and use alkalies at bedtime. A teaspoonful of bi-carbonate of soda on going to bed is a good prophylactic, and will often give relief if taken in the night when the cramp is felt. A pill of assafœtida at bedtime is also likely to keep off the cramp. When the cramp persists in the calf of the leg, stand on your feet and it will usually cease.

### COLLODION PAINTS.

R—Collodion.............................. oz. j.

Olei Palmæ...................... gtt. xxiv.

Anchusæ Radicis sufficient to give color.

A good artificial varnish or artificial cuticle in cutaneous abrasions, burns, etc.

### INFLAMMATION OF THE OS, OR IN VAGINISMUS.

*Conservative Treatment.*—Let the patient use the warm both by sitting in a tub of warm water for 20 minutes, with a small speculum inserted. On leaving the bath and before removing the speculum, insert a pledget of cotton the size of a walnut up against the os, well saturated with the following preparation:

Glycerin.................................. oz. j.

Carbolic Acid.............................. gtt. v.

Tannin.................................. gr. x.

Morphia.................................. gr.

A string should be attached to the pledget that it may be removed next morning. The treatment must be long continued to effect a cure. The morphine should not be used in the preparation unless there is considerable pain or restlessness at night.

### INTER UTERINE INJECTIONS WITHOUT THE USE OF SPECULUM.

A medical friend informs us that he has succeeded well in injecting, and in cauterizing the interior of the womb and the neck, by the use of the metallic male catheter applied by the touch with the finger of the left hand upon the os as a guide, and inserting the instrument to any required depth, and forcing the fluid through the catheter by means of a small syringe from without. Care should be taken not to use too much force, otherwise the fluid may enter the fallopian tubes and occasion peritonitis. W.

### TO MAKE ICE WATER.

Tosselli's method of cooling water consists of a cylindrical cup for holding the water; into this another vessel is plunged shaped like an inverted truncated cone, and having a projecting margin which rests on the outer cup. Four or five ounces of the nitrate of ammonia is added to the water in the inner cup, the solution of which is attended by a wonderful extraction of caloric, by which the water is converted into ice in a short time. The inventor claims to know another salt which will reduce the temperature 50 degrees Fahr.

### LARYNGEAL AFFECTIONS AND CATARRH.

Dr. George H. Rice, of St. Louis, Mo., writes:

JANUARY 14, 1878.

*Editor Southern Medical Record:*

If the enclosed "treatment" will be of any service to the many readers of your valuable journal, I herewith submit it, for laryngeal affections and old chronic catarrh. I received my patient on the first of December last; put him on an alterative for four weeks, and at the same time used the following new remedies:

R.—Pot. Iodide.......................grs. xxxv.
Grindelia Robusta...................oz. jss.
Yerba Santa.........................oz. jss.
Penthorum Sedoidee..................oz jss.

Syrup wild cherry, q s. to make an 8 oz. mix., and give a teaspoonful four times per day. It has proved a valuable remedy. The patient says he could not breath through both nares for the last six years; at the same time, also, his voice was husky, and now is all right, feeling entirely a different man. I shall continue the treatment in less doses until spring.

### DELIRIUM TREMENS.

The approved methods of treating delirium tremens may be sumarized as follows.

"Critical sleep to be brought about as soon as possible. Ice to cool irritable stomach. Salines, milk, raw eggs, beef tea, brandy and egg mixture, ammonia and bitters, ether, brandy and bark, sumbul and hop, bromide of potassium, morphia, chloroform and Indian hemp; Indian hemp in doses of half a grain to one grain. Subcutaneous injection of morphia; chloral, tincture digitalis in half ounce doses—(dangerous.) Cold affusions or cold shower bath sometimes useful. Avoidance of stimulation, and excessive doses of opium to be avoided, etc."—*Tanner.*

### CHEAP TONIC.

R—Chinoidine.........................oz. ss

Ac. Acitic...........................oz. ss

Water................................oz. xii

Ft. Sol. Dose, one teaspoonful.—*Med. & Surg. Rep.*

### FOR TAPE-WORM.

Leave off dinner and supper: eat a large onion at bed-time. Three hours before breakfast next morning take

Pulv. Kooso..........................dr. v

If it fail to operate in two hours take a large dose of oil and turpentine. The kooso should be mixed in half a teacupful of peppermint water. If it fails to bring the worm it will be because the article is not good.

When the kooso nauseates the stomach, it should be given in broken doses, repeated every hour until at least dr. v are taken.

Dr. Clark, of France prescribes the kooso as follows:

R—Pulv. Kooso........................dr. vij

Olei ricini (hot)....................oz. jss

Strain and pour in the residuum.

Aquæ bullietis.......................oz. jss

Filter and combine the two percolates by means of yolk of egg in an emulsion, and add gtt. xl, ether sulphuric, sweeten and aromatize, for one dose.—*Naphey.*

### VERMIFUGES.

R—Calmel.............................gr. vi
Santonin.............................gr. iij

Make three powders. Give one morning, noon and at bed-time in a little syrup. Next morning early give a dessertspoonful of castor oil and ten drops oil of turpentine to a child two years old.

Another:

L—Santonin...........................gr. iij
Fluid Ext. Pinkroot..................dr. ij
Syrup................................dr. ij

M. Give a teaspoonful three times a day until all is taken, and work off with castor oil. For a child three to five years old.

### WHOOPING COUGH.

Dr. Vogelsany, of Switzerland, finds that one or twe scruples of bromine and as much bromide of potassium to a glass of hot water, placed in the room of a child suffering from whooping cough affords it great relief. The mixture to be renewed three or four times a day.—*Naphey.*

### EARLY RUPTURE OF MEMBRANES.

Mr. Plaister, an English Surgeon, reports to a foreign journal 800 cases of midwifery, and claims that his experience establishes the propriety of rupturing the membranes in all cases when the os is the size of a shilling. We regard this man, and others of his class, who seek to improve upon nature's processes by uncalled-for interference, as dangerous men, whose advice should not be followed, howevei plausable their assertions or however great their claims to success. "Meddlesome midwifery is bad."

### OVARIAN TUMORS.

The muriate of ammonia has been found beneficial in ovarian enlargement. It is a good remedy also in uterine engorgement and in neuralgic affections connected with ovarian or uterine disorders. The following formula is specially suited to ovarian tumors:

R—Muriate Ammoniæ .................... oz. ss

Aquæ ................................ oz. xii

Tinc. Iodini ......................... dr. j

M. One tablespoonful three times a day.

### SORE NIPPLES.

We have found no application equal to the following for sore nipples. It is simple, easy of preparation, safe for the baby, and if applied when the child's mouth is sore, either from thrush or apthæ, it is a useful remedy for the child:

R—Pulv. acacioe

Sodæ biboratis .................. aa—oz ss.

M. Sprinkle a little upon the nipple immediately after the child is done sucking. W.

### SYPHILITIC RHEUMATISM.

For syphilitic rheumatism or nocturnal headaches in syphelitic subjects, use the following:

R—Iodide potassium .................. oz. ss

Com. Tinc. Gentian ................... oz. vi

S. Teaspoonful three times a day.

Another:

R—Muriate Ammonia .................... oz. j

Water ................................ oz. xij

Tinc. Iodine ......................... dr. ij

M. Teaspoonful threc times a day.

### MORTALITY IN PHILADELPHIA.

The number of deaths in Philadelphia during the year 1877 were 16,003; of these there were carried off by

| | |
|---|---|
| Consumption | 2,349 |
| Cholera Infantum | 978 |
| Pneumonia | 840 |
| Marasmus | 752 |
| Convulsions | 747 |
| Typhoid fever | 550 |
| Dropsy | 457 |

### MEMBRANOUS CROUP.

Dr. Booth, in *Med. & Surg. Rep.*, claims to have cured a case of membranous croup with the following:

R—Chlorate of potash .................. dr. ij
Syrup of lemon ....................... oz. j
Water ................................ oz. iij

Mix. Teaspoonful every hour. One dose of turpeth mineral was given, after which the chlorate mixture was given for several days.

### KEROSENE LINIMENT—CORRECTED.

The formula for this liniment, by a mistake of the printer, was improperly rendered in the last issue of the RECORD. It should read as follows:

R.—Kerosene Oil ....................... oz. ij.

Tinct. Opii .......................... dr. iv.

Tinct. Arnicæ ........................ dr. v.

Tinct. Stramonii ..................... dr. iv.

Spts. Ammon. Aromat .................. dr. vi.

Spts. Camphoræ ....................... dr. v.

Ol. Origani .......................... dr. iv.

Chloroform ........................... dr. iij.

M.

### FOR BRONCHITIS.

R—Carb. Ammoniæ ....................... gr. xv.

Spirits of Nitre ..................... dr. ij.

Syrup of Tolu.

Water ................................ aa—oz. i.

M. A teaspoonful every two hours for a child one or two years old, and double the quantity in the chronic coughs of adults, in debilitated conditions.

### QUININE FOR HYPODERMIC USE.

The following solution is used by English Surgeons hypodermically:

R—Quinæ disulphatis ................... gr. j

Acid. Sulphurici ..................... gtt. v

Acid Carbolici ....................... gtt. ij

Aequæ distillat ...................... dr. j

Inject thirty drops.

